# Ovary-derived circular RNAs profile analysis during the onset of puberty in gilts

**DOI:** 10.1186/s12864-021-07786-w

**Published:** 2021-06-15

**Authors:** Xiangchun Pan, Wentao Gong, Yingting He, Nian Li, Hao Zhang, Zhe Zhang, Jiaqi Li, Xiaolong Yuan

**Affiliations:** 1 Guangdong Laboratory of Lingnan Modern Agriculture, National Engineering Research Center for Breeding Swine Industry, Guangdong Provincial Key Lab of Agro-Animal Genomics and Molecular Breeding, College of Animal Science, South China Agricultural University, 510642 Guangzhou, China; 2 Guangdong Provincial Key Laboratory of Laboratory Animals, Guangdong Laboratory Animals Monitoring Institute, 510260 Guangzhou, China

**Keywords:** Alternative splicing, CircRNAs, Ovary, Puberty

## Abstract

**Background:**

In mammals, the ovary is the essential system of female reproduction for the onset of puberty, and the abnormal puberty has negative outcomes on health. CircRNA is a non-coding RNA produced by non-canonical alternative splicing (AS). Several studies have reported that circRNA is involved in the gene regulation and plays an important role in some human diseases. However, the contribution of circRNA has received little known within the onset of puberty in ovary.

**Results:**

Here, the profiles of ovarian circRNAs across pre-, in- and post-pubertal stages were established by RNA-sEq. In total, 972 circRNAs were identified, including 631 stage-specific circRNAs and 8 tissue-specific circRNAs. The biological functions of parental genes of circRNAs were enriched in steroid biosynthesis, autophagy-animal, MAPK signaling pathway, progesterone-mediated oocyte maturation and ras signaling pathway. Moreover, 5 circRNAs derived from 4 puberty-related genes (*ESR1, JAK2, NF1 and ARNT*) were found in this study. The A3SS events were the most alternative splicing, but IR events were likely to be arose in post-pubertal ovaries. Besides, the circRNA-miRNA-gene networks were explored for 10 differentially expressed circRNAs. Furthermore, the head-to-tail exon as well as the expressions of 10 circRNAs were validated by the divergent RT-qPCR and sanger sequencing.

**Conclusions:**

In summary, the profiles of ovarian circRNAs were provided during pubertal transition in gilts, and these results provided useful information for the investigation on the onset of puberty at the ovarian-circRNAs-level in mammals.

**Supplementary Information:**

The online version contains supplementary material available at 10.1186/s12864-021-07786-w.

## Background

Puberty is usually defined as the first estrus of mammals [1]. In human, the abnormal puberty has negative effects in diseases such as asthma [2], psychosocial disorder [3], hypogonadism [4] and reproductive system tumors [5, 6]. It has been well recognized that the initiation of puberty is mainly driven by the hypothalamus-pituitary-ovary (HPO) axis [7–9]. Several studies have shown that it is possible to treat abnormal puberty by regulating HPO axis [10–13] Furthermore, the ovary is the female reproductive system and the endocrine organ, which produces the steroids and peptide hormones necessary for the onset of puberty [14–16]. Previous studies have reported that the female puberty failure is presented with the decreased ovarian weight and denormal hormone levels in mice [17] and human [18]. Accumulating studies support that the noncoding RNA (ncRNAs) play a vital role in the expression of essential genes that regulate the oocyte growth and ovarian endocrine function [19, 20] as well as the onset of puberty [21, 22].

Circular RNAs (circRNAs) are the category of ncRNA molecules in the cytoplasm of eukaryotes, which are produced by non-canonical alternative splicing (AS) named back-splicing [23]. It is reported that most circRNAs are composed of only exonic sequences, while a few circRNAs are composed of the exon-intronic or intronic sequences [24–26]. More recently, thousands of circRNAs are identified through the high-throughput RNA sequencing (RNA-seq). In mammals, it was found that circRNAs are tissue-specific and stage -specific as well as evolutionarily conserved [27–30], and it has been shown that exonic circRNAs show miRNA sponge activity, and intronic circRNAs are likely to regulate the transcription of their host genes [31, 32]. These findings suggest that circRNAs may play a pivotal role in growth and development of mammals.

Recently, circRNAs have been found to involve in asthma [33, 34] and reproductive system tumors [35], both of which are closely related to the abnormal puberty, and circRNAs are reported to involve in oocyte maturation and hormone synthesis [36]. For example, Cao et al. showed that circRNAs derived from oocytes exhibit with the characteristics of developmental stage-specific expression [37]. Xin et al. revealed that the depletion of *circLDLR* inhibits the expression of *CYP19A1*, thereby reducing the secretion of estrogen in polycystic ovary syndrome [38]. Moreover, Jia et al. found that overexpression of *circEGFR* increases the production of estradiol, while knockdown of *circEGFR* enhances the production of progesterone in mice [39]. These observations suggested the potential importance and significance of circRNAs in the ovary, but the information of ovarian circRNAs remains little during the pubertal transition.

Collectively, in order to obtain more insights on the roles of circRNAs in ovaries during pubertal transition, the genome-wide analysis of circRNAs in ovaries across pre-, in- and post-puberty was performed by RNA-sEq. Then, the expression changes of circRNAs were explored as well as the stage-specific and tissue-specific circRNAs, and the potentially pubertal circRNAs were detected in this study. On the other hand, our study may provide a novel theoretical reference for the regulation of pubertal female by circRNAs.

## Results

### Identification of ovary-derived circRNAs during the onset of puberty

**Fig. 1:**
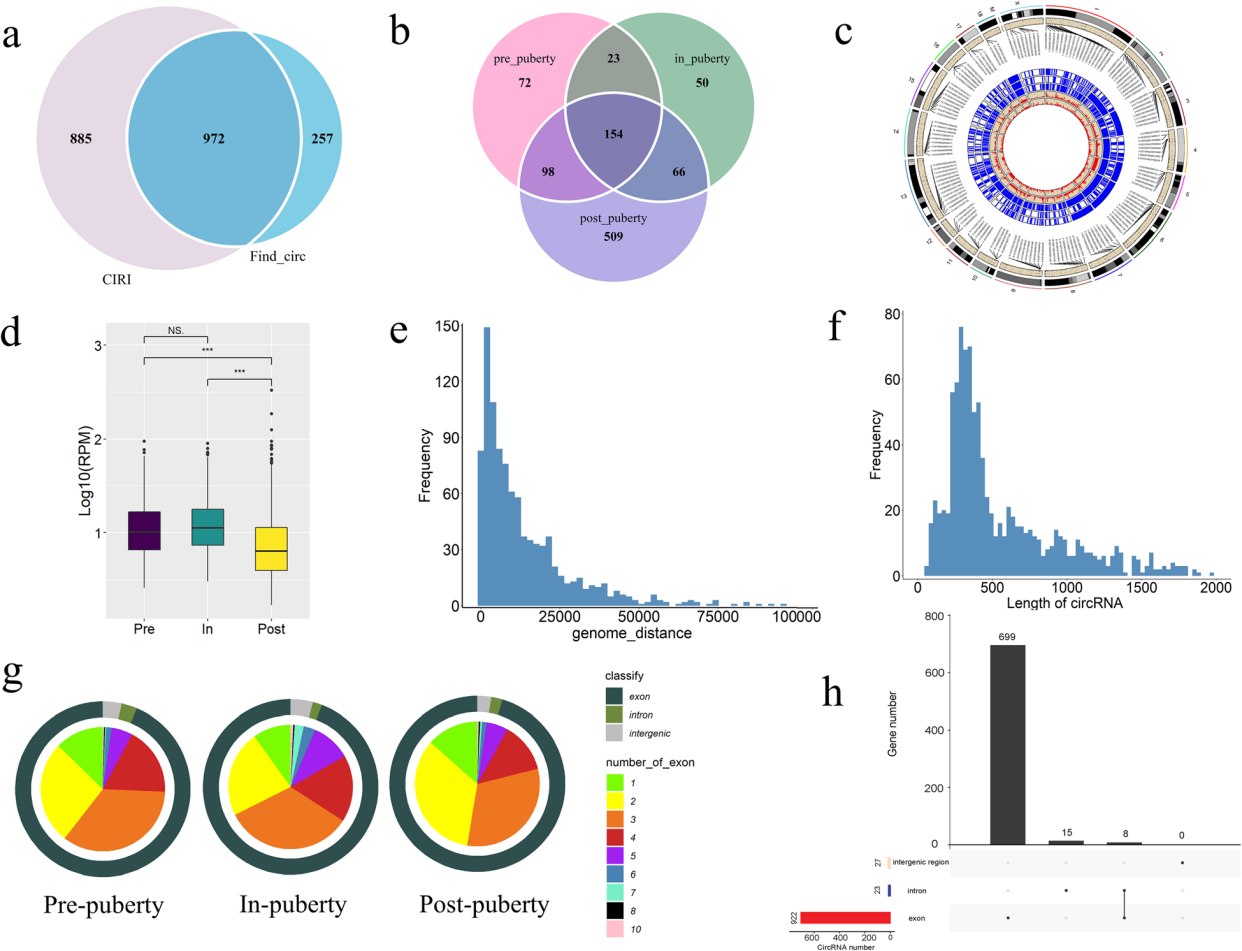
Overview of the identified circRNAs by RNA-seq analyses in ovaries of gilts. **a **CircRNAs were identified by two algorithms. **b** The Venn diagram shows the number of unique and common circRNAs in pre-, in- and post-puberty. **c** Circos plot of the circRNAs distribution in the whole genome of gilts. The outermost circle represents the distribution of the number of circRNAs, the blue circle represents the distribution of expression level of circRNAs, and the red inner circle represents the length of circRNAs. **d** Expression level of circRNAs in three stages, *p < 0.05. **e **Genomic dance of all detected circRNAs. **f** Transcript length of circRNAs.** g** Proportion of three types and exon number of the circRNAs in three stages. **h** The upset plot of three types of circRNAs and the corresponding parental genes

To obtain a reliable result, CIRI2 and find_circ software were intersected to identify circRNAs. A total of 972 circRNAs candidates were identified in the pubertal transition of pubertal ovaries (Additional file 1) (Fig. 1a). Thereinto, 347, 293 and 827 circRNAs were identified in pre-, in- and post-puberty, respectively (Fig. 1b). These circRNAs were more distributed in Sus scrofa chromosome 1 (Fig. 1c). The expressions of circRNAs were the lowest in the post-puberty (Fig. 1d), compared to pre- and in-puberty. Meanwhile, the average genome distance of all circRNAs was 13,453 bp, and circRNAs shorter than 50,000 bp were accounted for 96.7 % (Fig. 1e). Besides, the average length of all circRNAs was 558 bp, and circRNAs with the length of 200–500 bp were accounted for 53.70 % (Fig. 1f). Notably, in the pre-puberty, exonic, intronic and intergenic circRNA occupied 93.66 %, 3.46 and 2.88 %, respectively; in the in-puberty, exonic, intronic and intergenic circRNA occupied 94.20 %, 4.10 and 1.70 %, respectively; in the post-puberty, exonic, intronic and intergenic circRNA occupied 95.41 %, 2.54 and 2.05 %, respectively (Fig. 1 g). Additionally, most circRNAs were made up of two and three exons. Specifically, circRNAs were made up of two exons occupied 26.80 %, 22.53 and 33.98 % in pre-, in- and post-puberty, respectively; circRNAs were made up of three exons occupied 34.87 %, 33.45 and 31.56 % in pre-, in- and post-puberty, respectively (Fig. 1 g). Notably, 722 genes were identified as the parental genes of these 972 circRNA. 922 exonic circRNAs were derived from 699 genes, and 23 intronic circRNAs were derived from 23 genes (Fig. 1 h). Taken together, 972 circRNAs were identified during onset of puberty in the ovaries of gilts.

### Key pathways of cirRNAs in pubertal transition and circRNAs in pubertal genes

**Fig. 2:**
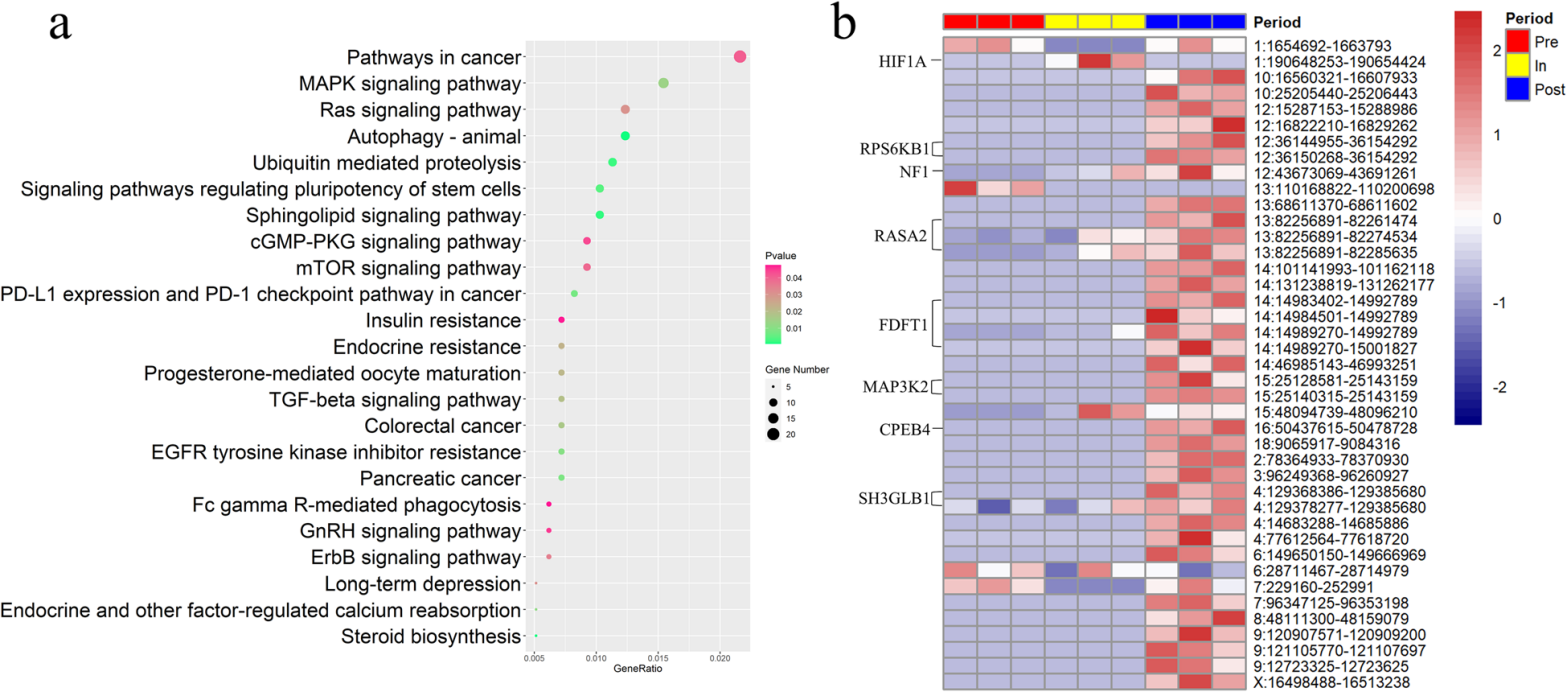
The key signaling pathway of cirRNAs in pubertal transition. **a** KEGG analysis of all identified circRNAs (*P < 0.05). **b** Expression level of circRNAs involved in pubertal key pathways in three stages

To further investigate the biological functions of circRNAs involved in pubertal ovary, the parental genes of all 972 circRNAs in puberty were used to the analysis of KEGG pathway (Additional file 2). Obviously, the functional pathways were significantly over-represented in pubertal ovaries, including steroid biosynthesis, autophagy-animal, MAPK signaling pathway, progesterone-mediated oocyte maturation and ras signaling pathway **(**Fig. 2a). In the steroid biosynthesis signaling pathway, “circ 14:14983402–14992789”, “14:14984501–14992789” and “14:14989270–15001827” derived from *FDFT1* gene were uniquely expressed in the post-puberty (Fig. 2b). In the autophagy-animal signaling pathway, “circ 1:190648253–190654424” derived from *HIF1A* gene was uniquely expressed in the in-puberty. In the MAPK signaling pathway, “circ 15:25128581–25143159” and “circ 15:25140315–25143159” derived from *MAP3K2* gene was uniquely expressed in the post-puberty. In the progesterone-mediated oocyte maturation, “circ 16:50437615–50478728” derived from *CPEB4* gene was uniquely expressed in the post-puberty. In the ras signaling pathway, “circ 12:43673069–43691261” derived from *NF1* gene was expressed in the in-puberty and post-puberty. Details of these circRNAs were shown in Additional file 3. Moreover, in order to further explore the circRNAs in pubertal genes, 10 puberty-related genes (*ESR1*, *JAK2*, *NF1*, *ARNT*, *IGF1*, *KISS1*, *Gpr54, NKB, Mkrn3*, *GnRH*) were selected and explored by manual reviewing the literature and databases, and 5 circRNAs derived from 4 pubertal genes (*ESR1*, *JAK2*, *NF1*, *ARNT*) were lastly found in the ovary of pubertal transition (Additional file 4). “circ 1:14373232–14374308” (uniquely expressed in the pre-puberty) and “circ 1:14416335–14457143” (expressed in the pre-, in- and post-puberty) were derived from *ESR1*; “circ 1:216914275–216951002” (uniquely expressed in the post-puberty) was derived from *JAK2*; “circ 12:43673069–43691261” (expressed in the in- and post-puberty) was derived from *NF1*; “circ 4:98369520–98372553” (uniquely expressed in the post-puberty) was derived from *ARNT*. Apart from “circ 1:14416335–14457143” and “circ 12:43673069–43691261”, other 3 circRNAs derived from 4 pubertal genes showed stage-specific expressions.

### AS of circRNAs in gilts’ ovaries during puberty

**Fig. 3:**
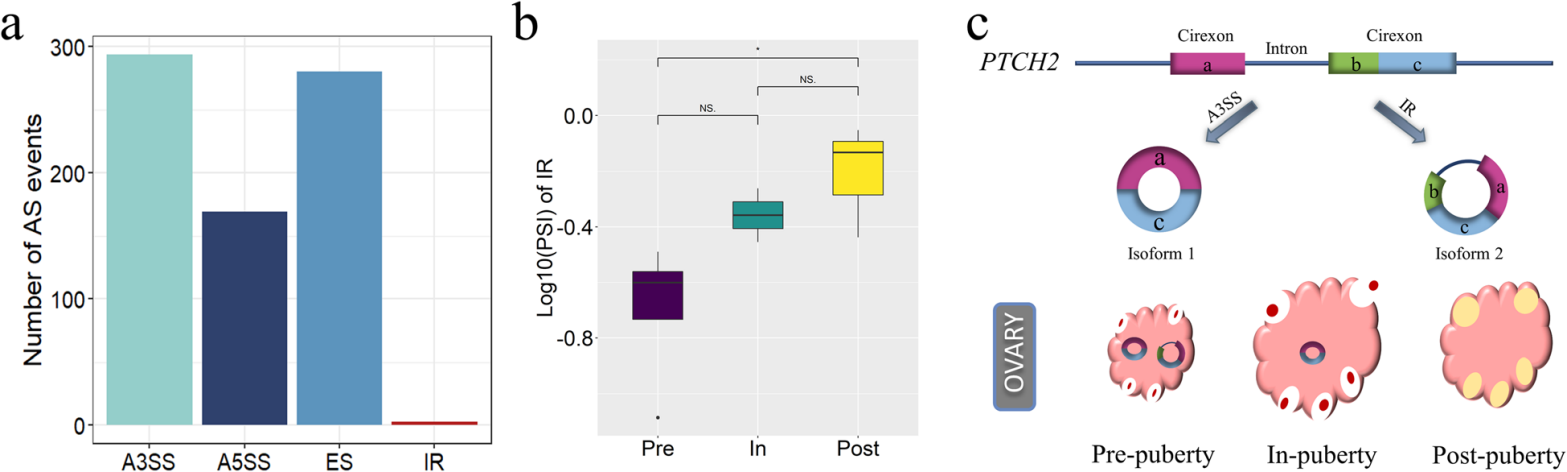
The alternative splicing (AS) events of circRNAs and the presumed formation of cirRNAs in pubertal transition. **a** Number of four types of AS events of all detected circRNAs. **b** Differential IR events with the value of PSI value in three stages, *p < 0.05. **c** Two isoforms of circRNAs might were derived from PTCH2 by A3SS and IR splicing patterns

The formation of circRNAs is dependent on AS [40]. In order to further explore the AS events involved in circRNAs, we identified the splicing events in circRNAs. Compared with other events, A3SS events were the most splicing pattern in ovaries of gilts in puberty (Welch two-sample t-test, *P* < 0.05) (Fig. 3a). Strikingly, in the four types of AS events, IR showed more extreme post-pubertal tendency (Welch two-sample t-test, *P* < 0.05) (Fig. 3b). In other words, there were difference in IR event between pre-puberty and post-puberty. Furthermore, “circ 6:166505226–166505778” existed two isoforms, and its parental gene (*PTCH2*) was reported to regulate the follicle development [41] (Additional file 5). The isoforms spliced by A3SS events exist in pre- and in-puberty, but do not exist in post-puberty; the isoforms spliced by IR events exclusively exist in pre-puberty (Additional file 5) (Fig. 3c). The results above showed that the AS events might play a crucial role in formation of ovarian circRNAs during puberty.

### Stage-specific and ovary-specific circRNAs in the pubertal transition

**Fig. 4:**
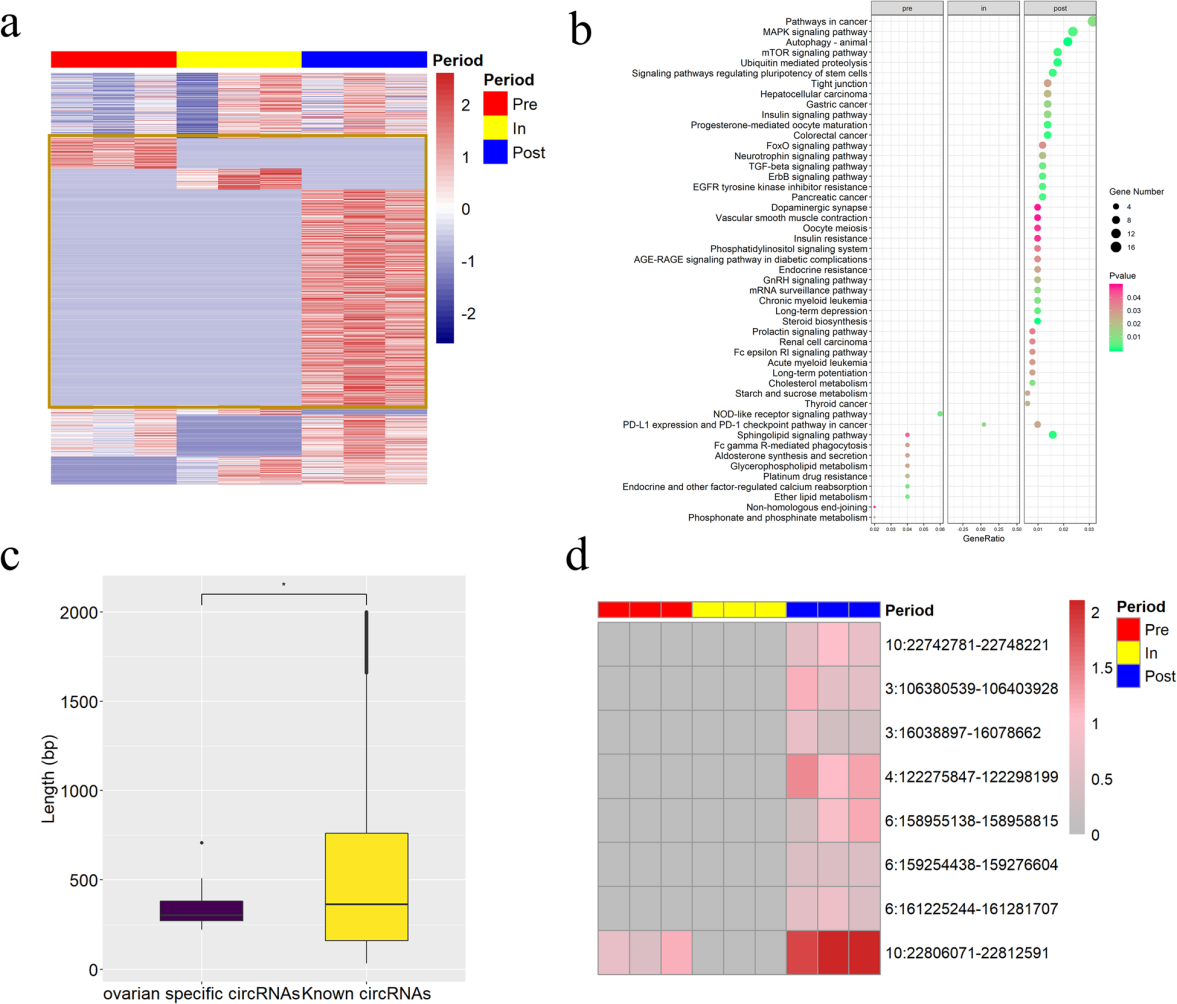
Analysis results of stage-specific and ovary-specific circRNAs. **a** Expression level of all circRNAs in three stages. **b** KEGG analysis of the parental genes of stage-specific circRNAs (P < 0.05). **c** Length of ovary-specific circRNAs and known circRNAs, *p < 0.05. **d** Expression level of ovary-specific circRNAs in three stages

To further explore the stage-specific circRNAs during these pubertal stages, we investigated the expression of circRNAs in pre-, in- and post-puberty stages. Respectively, 72, 50 and 509 of circRNAs were uniquely expression in pre-, in- and post-puberty stages and considered to be stage-specific circRNAs (Fig. 4a). The parental genes of these stage-specific circRNAs were enriched in MAPK signaling pathway, progesterone-mediated oocyte maturation, oocyte meiosis and GnRH signaling pathway in post-puberty (Additional file 6) (Fig. 4b). Moreover, 154 circRNAs were expressed in all stages and considered as the co-existed circRNAs (Figs. 1b and 4a). Besides, some specific circRNAs and co-existed circRNAs were derived from the same gene. For instance, “circ 1:100589850–100603238” (uniquely expressed in the post-puberty) and “circ 1:100589850–100613174” (no uniquely expressed in any puberty) were derived from *SMAD4* (Additional file 7). In order to further investigate the specific circRNAs in the ovaries, 964 known circRNAs that were found in nine tissues (brain, heart, kidney, liver, lung, skeletal muscle, spleen, testis, and retina) were excluded, leaving 8 circRNAs as being putative ovary-specific circRNAs which were only expressed in the ovary (Additional file 8). Subsequently, we found that the length of ovary-specific circRNAs were shorter than known circRNAs (Fig. 4c). Strikingly, apart from “circ 10:22806071–22812591”, other 7 putative ovary-specific circRNAs were exclusively expressed in the post-puberty (Fig. 4d). Besides, “circ 10:22806071–22812591” and “circ 10:22742781–22748221” were both derived from *NR5A2* (Additional file 8). To sum up, the results showed that 64.92 % (631/972) of ovarian circRNAs expressed in the stage-specific means during pubertal transition, and 8 circRNAs were the ovary-specific circRNAs.

### Potentially regulated network of differentially expressed circRNAs

**Fig. 5:**
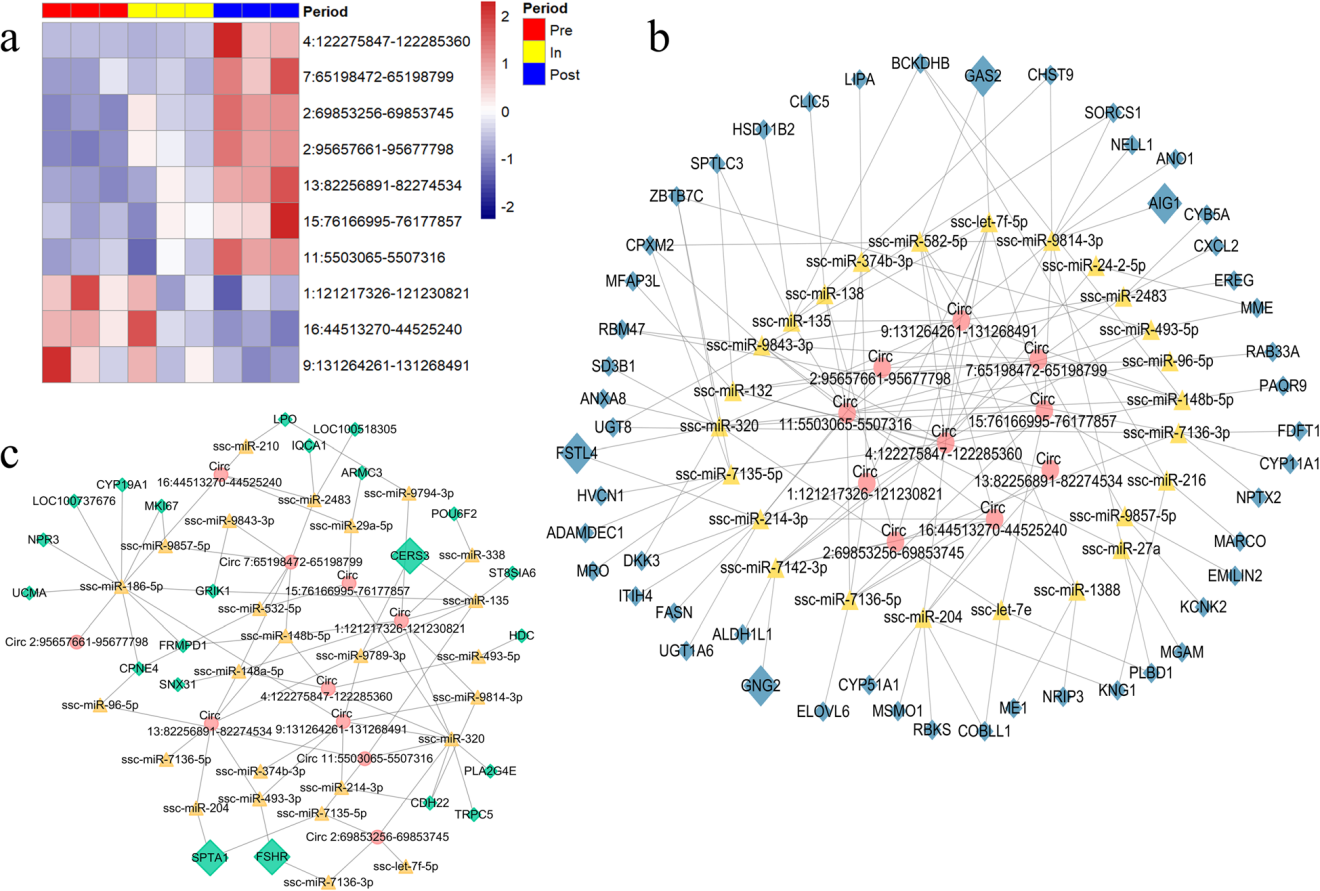
The potentially regulatory network for differentially expressed circRNAs. **a** Expression level of differentially expressed circRNAs in three stages. **b** Potentially network of differentially regulated circRNAs with up-regulated mRNAs. **c** Potentially network of differentially regulated circRNAs with down-regulated mRNAs. The red circle represents circRNAs, the yellow triangle represents miRNAs, the blue diamond represents up-regulate gene, the green diamond represents down-regulate genes

Subsequently, 154 co-existed circRNAs were used to analysis differential expression between pair-wise comparison of three stages (Figs. 1b and 4a). In total, 10 circRNAs were identified as differentially expressed circRNAs (Additional file 9), of which 7 up-regulated circRNAs and 3 down-regulated circRNAs were identified in the pre- vs. post-puberty group; 2 up-regulated circRNA were identified in the in- vs. post-puberty group (Fig. 5a). To further explore the possible functions of the differentially expressed circRNAs, we tried to predict the miRNA binding sites of these circRNAs and explore the possible relationship between differentially expressed circRNAs and differentially expressed genes. The miRNAs with the top 5 highest score in miRanda-based circRNAs match were selected as potential miRNA targets and listed in Additional file 9 (see Methods for further details). We found that the differentially expressed circRNAs might interact with many of miRNAs, or might indirectly interact with differentially expressed genes (Fig. 5b-c). Noticeably, we also highlighted *FSTL4*, *GAS2*, *AIG1*, *GNG2*, *FSHR* and *SPTA1* genes, which were associated with folliculogenesis or hormone [42–47]. For instance, “circ 9:131264261–131268491”, which was down-regulated in the pre- vs. post-puberty groups, might interact with *FSTL4* via ssc-miR-320, might interact with *GAS2* via ssc-miR-582-5p, and might interact with *FSHR* via ssc-miR-493-3p (Additional files 9 and 10). Taken together, the circRNA-miRNA-gene networks were explored for 10 differentially expressed circRNAs.

### Validation of circRNAs

**Fig. 6:**
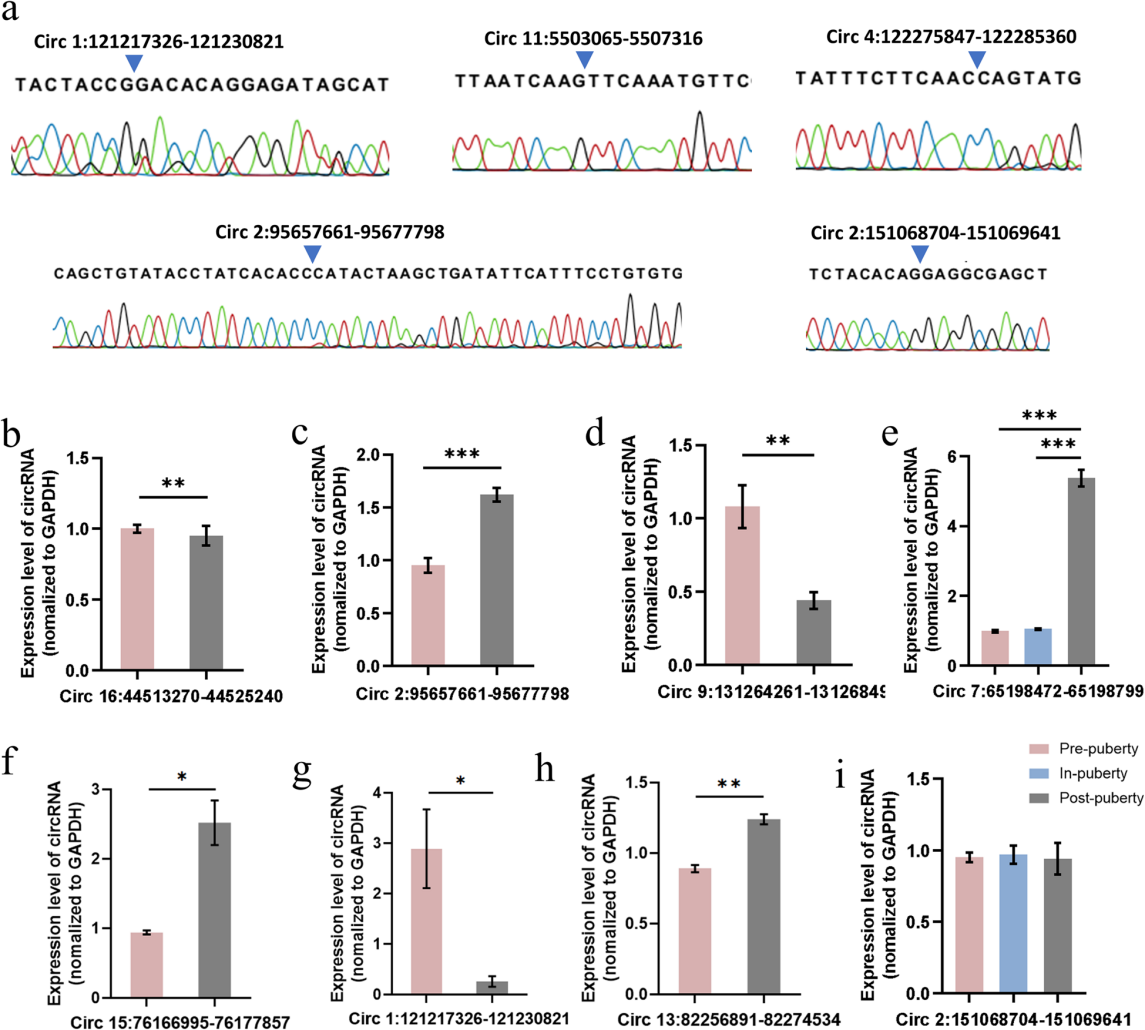
Sanger sequencing and RT-qPCR validation of circRNAs. **a** sanger sequencing of five circRNAs showed the back-splice junction. **b-h** seven circRNAs of differential expression and **i** one circRNA of insignificant difference was randomly selected for RT-qPCR. The primer information was listed in Additional file 11, *p < 0.05, **p < 0.01, *** p < 0.001

To verify the accuracy of our data, the divergent RT-PCR and sanger sequencing were utilized to identify the authenticity of circRNA, the head-to-tail splice junctions, as well as the expressions of circRNAs. The head-to-tail splice junctions of 5 circRNAs were determined by sanger sequencing, which proved that the circRNAs were circRNAs (Fig. 6a). Furthermore, 7 circRNAs of 10 differentially expressed circRNAs were selected for further investigation. The “circ 7:65198472–65198799” (Fig. 6b), “circ 16:44513270–44525240” (Fig. 6c), “circ 2:95657661–95677798” (Fig. 6d), “circ 9:131264261–131268491” (Fig. 6e), “circ 15:76166995|76177857” (Fig. 6f), “circ 1:121217326|121230821” (Fig. 6 g), and “circ 13:82256891|82274534” (Fig. 6 h) were significantly differentially expressed, which are in line with the RNA-sEq. Besides, the expression of “circ 2:151068704–151069641”, which was detected insignificantly differentially expressed, was insignificantly changed (Fig. 6i). Finally, results showed that the expressions of 8 selected circRNAs verified by divergent RT-qPCR were consistent with the trend of RNA-seq data (Additional file 9). Our results indicated that the existence of differentially expressed circRNAs, which further shows that our analysis is reliable.

## Discussion

In mammals, the onset of puberty is highly implicated in reproduction, and the abnormal puberty can cause various diseases. For instance, the precocious girls have twice the risk of asthma in adulthood than normally pubertal girls [2]. We have previously demonstrated that pituitary-specific circRNAs are related to reproduction-associated signaling pathways in pubertal gilts [48]. The ovary, as the important member of HPO axis, has been reported to guide female into puberty. Zhao et al. showed that *circ_0023942* might inhibit the proliferation of human ovarian granulosa cells by regulating the expression of *CDK-4* [49]. Therefore, it is necessary to profile the expressions and changes of circRNAs in ovaries during pubertal transition. In this study, the circRNAs we obtained are widely distribution on 1 chromosome, which is consistent with previous literatures [48, 50]. It is worth noting that previous report has shown that circRNAs are divided into three categories, of which exonic circRNAs account for the majority [50]. Consistently, in this study, we demonstrated that exonic circRNAs were accounted for approximately 94 %.

Moreover, we uncovered several circRNAs in some of the key pathways associated with the onset of puberty, including steroid biosynthesis, autophagy-animal, MAPK signaling pathway, progesterone-mediated oocyte maturation and ras signaling pathway. These pathways have all been reported to regulate the development of ovary and onset of puberty [41, 51–53]. Notably, for autophagy-animal pathway, “circ 1:190648253–190654424” was exclusively expressed in the in-puberty, and its parental gene *HIF1A* has been reported to have the highest expression in the early luteal phase [54]. Therefore, circRNA “circ 1:190648253–190654424” might play a crucial role in autophagy in puberty. Furthermore, we found that 5 circRNAs derived from *ESR1, JAK2, NF1* and *ARNT*, which are associated with the onset of puberty [55–58]. Previous studies have shown that circRNAs can promote the transcription of their parental genes [24, 26]. It is possible therefore that these 5 circRNAs might contribute to the onset of puberty by promoting transcription of *ESR1, JAK2, NF1* and *ARNT*.

Interestingly, AS not only formed the linear mRNA, but also formed circRNAs, which caused the diversity of circRNAs [40]. For instance, compared with linear mRNAs, exons in Exon Skipping (ES) events were more likely included in circRNAs [26], and some introns are retained in circRNAs through alternative splicing [25]. In this study, we found that the AS events in circRNAs were consistent with previous reports, of which A3SS and ES events occurred in circRNAs in abundance. Furthermore, circRNA “6:166505226–166505778” from *PTCH2* gene was spliced by IR and A3SS events in pre-puberty, while by A3SS in in-puberty (Fig. 3). It has been reported that *PTCH2* is related to the functions of granulosa cells in ovaries of mammals [59, 60]. It is possible that AS events were differentially expressed in three pubertal stages.

Previous studies have shown that circRNAs have stage-specific and tissue-specific expression patterns [37]; Xia et al. comprehensively analysis circRNAs in human and mouse to establish a tissue-specific circRNAs database [27]; G. Maass et al. have shown that circRNAs exhibit a high degree of tissue-specificity in clinically relevant human tissues [61]. According to our analysis of stage-specific circRNAs during the pubertal transition in gilts, we found that 509 of circRNAs specifically existed in post-puberty. Importantly, the parental genes of post-pubertal specific circRNAs were associated with the function of growth, development and reproduction. These results indicated that circRNAs might influence parental genes to regulate the puberty in female of mammals. Moreover, the parental genes of 8 circRNAs that were found uniquely expressed in ovaries might regulate the growth and development of ovary. For example, the parental gene of “circ 10:22806071–22812591” was *NR5A2*; the parental gene of “circ 6:161225244–161281707” was *FAF1*; the parental gene of “6:158955138–158958815” was *LRP8*. Guo et al. have shown that enhancing the expression of *NR5A2* stimulates the progesterone synthesis in granulosa cell of porcine [62]. There is evidence that *FAF1*, Fas-associated protein factor 1, is expressed in the cytoplasm of growing oocyte of the ovary [63]. Yang et al. have provided that *LRP8* is the progestogenic-associated gene [64]. These results suggest that the tissue-specific circRNAs might regulate the transcriptions of their parental genes to involve in the onset of puberty.

Besides, many of target genes regulated by circRNAs-miRNAs network are the important puberty related genes and differentially expressed in the pubertal ovaries (Fig. 5b-c). For example, *GAS2* is a regulator in the ovary during folliculogenesis and oocyte cyst breakdown [43]; *AIG1* is induced by androgen and expressed at high levels in the ovaries [44]; *SPTA1* might be related to oocyte maturation [65]; F*SHR* is expressed in granulosa cells that regulates proliferation of granulosa cell, maturation of follicular and production estrogen [46]; *CERS3* is related to androgen production [66]. Studies have shown that circRNAs might act as a miRNA sponge to co-regulate the expression of related genes with miRNA [29]. For instance, *circPUM1* acted as a molecular sponge of mir-760 and reduced the expression of mir-760, which in turn affected polycystic ovary syndrome [67]. Moreover, Meng et al. characterized circRNA in healthy antral and atretic antral follicles, and found that abnormal circRNAs might play a crucial role in antral follicular atresia in pig [68]. Another study showed that *circINHA* promoted the proliferation of granulosa cells and inhibited the apoptosis of granulosa cells by acting as a miR-10a-5p sponge in pig [69]. In this study, we found that the differentially expressed genes interacted with differentially expressed circRNAs. For instance, in pre- vs. post-puberty group, the down-regulated “circ 9:131264261–131268491” interacted with the down-regulated *FSHR*, indicating that this circRNAs might as the miRNAs sponge to affect the expression of *FSHR*. These results suggested that circRNAs might affect the expression of genes by acting as sponge of miRNA, which in turn influence the onset of puberty in mammals. On the other hand, further investigations are needed to identify the functions of circRNAs.

## Conclusions

In conclusion, we described the profiles of ovarian 972 circRNAs during pubertal transition in gilts, and these circRNAs were mostly enriched in steroid biosynthesis, autophagy-animal, MAPK signaling pathway, progesterone-mediated oocyte maturation and ras signaling pathway. 631 circRNAs were stage-specific, 8 circRNAs were tissue-specific, and 10 circRNAs were differentially expressed across pre-, in- and post-pubertal ovarian. Besides, 5 circRNAs were derived from four pubertal genes *ESR1, JAK2, NF1* and *ARNT*. The isoforms of circRNAs spliced by IR were more likely to take place in post-puberty, and circRNA-miRNA-gene networks were explored for 10 differentially expressed circRNAs. Furthermore, several circRNAs were validated by the divergent RT-qPCR. These results suggest that circRNAs might play a crucial role in mammalian ovaries during onset of puberty, and further research should be undertaken to investigate the molecular mechanism of ovarian circRNAs in pubertal onset of mammals.

## Methods

### Preparation of samples

The gilts were purchased from Baishi Pig Farm, Zhongshan City, Guangdong Province, China. Three groups of Landrace × Yorkshire crossbred gilts were used: three gilts were served as pre-puberty gilts which were 160 days in age without any pubertal signs (weight = 81.38 ± 2.40 kg, age = 162 ± 3 d, no reddening, no swelling of the vulva, no standing reflex); three gilts were designated as the in-pubertal gilts which exhibited first pubertal signs (weight = 110.00 ± 2.00 kg, age = 212 ± 14 d, reddening, swelling of the vulva, standing reflex); three gilts were selected as the post-pubertal gilts which were 14 days beyond the pubertal phase (weight = 122.82 ± 9.11 kg, age = 216 ± 17 d). After euthanasia, the gilts’ ovaries (diameter ≥ 5 mm) were removed immediately to the liquid nitrogen, then stored at − 80 °C until further use.

### RNA sequencing and data processing

Pre-, in-, and post-pubertal gilts’ ovaries were extracted the total RNA using the Trizol agent (Invitrogen, Carlsbad, CA, USA), the total RNA quality was then measured using the Agilent Bioanalyzer 2100 system (Agilent, Palo Alto, California, USA). Filter RNA samples with RNA integrity values greater than 7.0, and remove rRNA from qualified total RNA using Epicentre Ribo-zero rRNA removal kits (Epicentre, Madison, WI, USA). We then used rRNA-depleted RNA to form a double-stranded cDNA with the mRNA-Seq sample preparation kit (Illumina, SanDiego, USA). Later, the cDNA library of each sample was sequenced using the HiSeq 2500 sequencer based on the manufacturer’s instructions and further produced 150 bp paired-end reads. These raw reads were used the Cutadapt software to remove the low-quality reads and the 3’ adaptor-trimming for the quality control [70]. The clean data that after quality controlled was then mapped with two software, which were BWA and bowtie2 software [71], and the reference genome used Sus scrofa11.1.

### CircRNA identification and data analysis

Reports showed that CIRI2 has high sensitivity and low FDR, thus we used CIRI2 identified circRNA after BWA [71], as well as using find_circ [72] to identify circRNA after bowtie2 to minimize the number of false positives. The two programs look for potential circRNAs based on genomic comparisons. We screened circRNA with at least 2 unique junction reads as candidates, removed circRNAs with unclear break point, and filtered circRNA with a length greater than 100 kb (genome length, which defined as the distance from the first exon to the last exon in the circRNA). We eventually identified candidate circRNA in the gilts during pre-, in- and post-puberty. Thereinto, CIRI2 generated 1-base coordinates, but find_circ generated 0-base coordinates, thus we converted the two coordinates into a consistent 1-base for later analysis. Subsequently, we set the circRNA detected only in one pubertal stage as a stage-specific circRNA. In addition, the selection criteria for tissular specificity was as follows: the circRNAs identified in this study were matched with the known circRNAs in pigs by starting and ending the genome locations of circRNAs, and the new circRNAs were considered as the presumed tissue specific circRNAs. The known circRNAs were downloaded from circAtlas 2.0 (namely, the circRNAs database in vertebrates) which were included circRNAs of nine tissues (brain, retina, heart, kidney, liver, lung, skeletal muscle, spleen, and testis) [73]. In addition, the alternative splicing events of circRNAs were determined by the CIRI-AS module [40], which classified the alternative splicing events into four forms: A3SS, A5SS, ES, and IR. The criteria for differential alternative splicing was as follows: PSI as the expression value, was subjected to the difference significance test (*t*-test) between any two pubertal pig groups. In this study, the EBSeq package was used to calculate the expression levels of circRNAs [74], which was quantified in RPM using the number of splicing junctions. The criteria for differentially expressed circRNAs was log2-fold_change- ≥ 1, adjusted p (p.adj) < 0.05. In addition, the value of any two pubertal pig groups was subjected to the difference significance test (Welch two-sample t-test) to analyze the significant differences.

### Prediction of miRNA target and circRNA-miRNA-mRNA network construction

The interaction of circRNA-miRNA-gene was predicted by miRanda software [75] with a miRanda match score ≥ 175. The specific method is as follows: all the miRNAs sequence of Sus scrofa was obtained from miRBase database (http://www.mirbase.org/), all the circRNAs sequence was obtained using Bedtools, and the match score of miRNA and circRNA was scored utilizing miRanda, miRNAs with top 5 matching scores were eventually predicted. Furthermore, Bedtools [76] was used to extract the differentially up-regulated and down-regulated mRNA sequences between any two pubertal pig groups (p.adj < 0.05, |log_2_FC| ≥3 or ≤ − 3), respectively. Subsequently, miRanda software was used to predict the target genes of miRNA according to these sequences. Finally, the interoperability between circRNA-miRNA-gene was then described by the cytoscape software [77].

### Pathway analysis

The parental gene of circRNA was analyzed by the KOBAS online software (http://kobas.cbi.pku.edu.cn/) with its GO function enrichment and KEGG pathway analyses [78]. The hypergeometric test significance threshold P < 0.05 was considered for genes to indicate significant enrichment.

### Validation of CircRNA

Back-spliced junction (BSJ) was a region consisting of canonical 5’ splice site sequence connected to upstream 3’ splice site sequence. The reliability of circRNA is usually verified by divergent primer flanking the BSJ utilizing RT and quantitative PCR (RT-qPCR) assays [29]. The divergent primers of 10 circRNAs were designed to verify the accuracy of the RNA-sEq. Firstly, in accordance with the operator’s procedures of Taq PCR MasterMix (Tiangen, China), the cDNA template PCR were amplified by 35 cycles at 95 °C (30 s), 60 °C (30 s) and 72 °C (20 s), and the PCR product was visualized using a 2 % GelRed-stained agar glycogel. Furthermore, the sanger sequencing was further performed to directly examine the PCR product. In accordance with the manufacturer’s protocol, PrimeScript RT Reagent Kit (TaKaRa, Osaka, Japan) in a Mx3005P real-time PCR System (Stratagene, La Jolla, CA, USA) was used to qPCR, and the PCR standard procedure was denaturation 94 °C (5 min), 40 cycles at 94 °C (10 s), 52 to 62 °C (15 s), and 72 °C (30 s). As an internal reference, GAPDH normalized expression of circRNAs. We used the 2^−ΔΔCt^ method to analyze the RT-qPCR data, and used the Student’s t test to assess differences in means of any two pubertal pig groups, the screening criteria for statistically significant were adjusted *p* < 0.05 (t-test). All of PCR primer were divergent design by the head-to-tail exon of circRNAs and listed in Additional file 11.

## Supplementary Information

**Additional file 1**. List of the information of all identified circRNAs.

**Additional file 2**. List of the KEGG pathways enriched using parental genes of all CircRNAs.

**Additional file 3**. List of the key pathways related to the timing of puberty in parental genes of circRNAs.

**Additional file 4**. List of the circRNAs in pubertal genes.

**Additional file 5**. List of the alternative splicing in circRNAs.

**Additional file 6**. List of the KEGG pathways enriched using parental genes of stage-specific CircRNAs.

**Additional file 7**. List of the parental genes that are capable of producing stage-specific and non-specific circRNAs.

**Additional file 8**. List of the tissue-specific circRNAs.

**Additional file 9**. List of the differentially regulated circRNAs.

**Additional file 10**. List of the differentially expressed genes associated with puberty our development of ovary.

**Additional file 11**. List of primers used for validation.

## Data Availability

The datasets used in this study have been submitted to the European Nucleotide Archive under accession number PRJEB39730 (https://www.ebi.ac.uk/ena/browser/view/PRJEB39730).

## Abbreviations

AS: Alternative splicing
circRNA: Circular RNA
HPO: Hypothalamus-pituitary-ovary
ncRNAs: Noncoding RNA
ES: Skipping exons
A3SS: Alternative 3´ splice site
A5SS: Alternative 5´ splice site
IR: Intron retention
PCR: Polymerase chain reaction
RT-qPCR: Quantitative real-time PCR
